# Uniform electroactive fibre-like micelle nanowires for organic electronics

**DOI:** 10.1038/ncomms15909

**Published:** 2017-06-26

**Authors:** Xiaoyu Li, Piotr J. Wolanin, Liam R. MacFarlane, Robert L. Harniman, Jieshu Qian, Oliver E. C. Gould, Thomas G. Dane, John Rudin, Martin J. Cryan, Thomas Schmaltz, Holger Frauenrath, Mitchell A. Winnik, Charl F. J. Faul, Ian Manners

**Affiliations:** 1 School of Chemistry, University of Bristol, Cantock’s Close, Bristol BS8 1TS, UK; 2 Bristol Centre for Functional Nanomaterials, University of Bristol, Tyndall Avenue, Bristol BS8 1TL, UK; 3 European Synchrotron Radiation Facility, BP 220, Grenoble F-38043, France; 4 Folium Optics Ltd, Unit 28, Cooper Road, Thornbury, Bristol BS35 3UP, UK; 5 Department of Electrical and Electronic Engineering, University of Bristol, Woodland Road, Bristol BS8 1UB, UK; 6 Laboratory of Macromolecular and Organic Materials, Institute of Materials, Ecole Polytechnique Fédérale de Lausanne (EPFL), EPFL–STI–IMX–LMOM, Station 12, 1015 Lausanne, Switzerland; 7 Department of Chemistry, University of Toronto, Toronto, Ontario, Canada M5S 3H6

## Abstract

Micelles formed by the self-assembly of block copolymers in selective solvents have attracted widespread attention and have uses in a wide variety of fields, whereas applications based on their electronic properties are virtually unexplored. Herein we describe studies of solution-processable, low-dispersity, electroactive fibre-like micelles of controlled length from π-conjugated diblock copolymers containing a crystalline regioregular poly(3-hexylthiophene) core and a solubilizing, amorphous regiosymmetric poly(3-hexylthiophene) or polystyrene corona. Tunnelling atomic force microscopy measurements demonstrate that the individual fibres exhibit appreciable conductivity. The fibres were subsequently incorporated as the active layer in field-effect transistors. The resulting charge carrier mobility strongly depends on both the degree of polymerization of the core-forming block and the fibre length, and is independent of corona composition. The use of uniform, colloidally stable electroactive fibre-like micelles based on common π-conjugated block copolymers highlights their significant potential to provide fundamental insight into charge carrier processes in devices, and to enable future electronic applications.

## Abstract (web summary)

The self-assembly of block copolymers to form micelles has been used in applications such as drug delivery and composite reinforcement. Here the authors explore the use of fibre-like micelles of controlled length in the active layer of field-effect transistor devices.

The self-assembly of block copolymers (BCPs) in solvents selective for one segment offers an important and well-developed method for the formation of spherical and cylindrical (or fibre-like) micelles and vesicles^1^. The resulting nanostructures show colloidal stability due to the presence of the solvent swollen corona and have been exploited for a variety of uses including the delivery of therapeutic cargoes in nanomedicine^2^, as additives for friction reduction^3^, fillers for composite reinforcement^4^ or for the formation of nanoscopic arrays using optical tweezers^5^. However, although recent advances have described examples of fibre-like micelles with novel photophysical characteristics^6^,^7^, their assembly into complex semiconducting particles, bundles and branched superstructures^8^,^9^,^10^,^11^, and their fabrication of coaxial nanowires with quantum dots^12^,^13^, ordered two-dimensional assemblies at interfaces^14^ and solar cells^15^, the evaluation of their potential as functional electroactive materials in devices is virtually unexplored^14^,^15^; specifically, uniform samples of low length dispersity have not been investigated.

As a consequence of their low density and solution processablity, π-conjugated semiconducting polymers possess many advantages over traditional semiconductors and metals, leading to their extensive exploitation in photovoltaics^16^,^17^,^18^, light-emitting devices^19^,^20^,^21^, field-effect transistors^22^,^23^,^24^ and sensors^25^,^26^. Fibres fabricated from solely π-conjugated homopolymers have been studied in particular detail and high charge carrier mobilities (*μ*) have been reported^27^,^28^,^29^. However, these one-dimensional nanostructures exhibit limited colloidal stability and access to uniform samples of controlled length that allows optimization for different applications remains a severe challenge^30^,^31^.

Herein we report the exploration of the potential of colloidally stable fibre-like BCP micelles with precisely controllable length, low dispersities and a π-conjugated core both to provide insight into the factors that influence device performance and also to enable wider organic electronic applications.

## Results

### Preparation of uniform fibre-like micelles

The BCPs used to prepare the fibre-like micelles used in this study possessed a crystallizable core-forming regioregular poly(3-hexylthiophene) (*rr*P3HT) block^6^,^7^,^8^,^9^,^10^ and a solubilizing, amorphous coronal block. To allow us to probe the influence of the corona on charge transport, two different coronas were investigated: regiosymmetric poly(3-hexylthiophene) (*rs*P3HT)^21^,^32^,^33^,^34^ and polystyrene (PS). The *rr*P3HT-*b*-*rs*P3HT materials were prepared via sequential Grignard metathesis (GRIM) polymerization (Supplementary Fig. 1a)^35^,^36^, whereas the *rr*P3HT-*b*-PS BCPs were synthesized by a Cu-catalysed azide–alkyne cycloaddition reaction, using azide-terminated PS and alkyne end-functionalized *rr*P3HT homopolymers^37^,^38^. The sequential GRIM polymerization has been extensively explored and has been widely used to synthesize thiophene-based all-conjugated BCPs.^9^,^10^,^35^,^36^ The characteristics of *rr*P3HT_106_-*b*-*rs*P3HT_47_, *rr*P3HT_48_-*b*-*rs*P3HT_43_, *rr*P3HT_54_-*b*-PS_44_, *rr*P3HT_70_-*b*-PS_197_ and homopolymer *rr*P3HT_48_ (the subscripts represent the degree of polymerization (DP) of each block) are summarized in Supplementary Table 1 and Supplementary Figs 1–3.

Uniform fibre-like micelles were fabricated from the *rr*P3HT-*b*-*rs*P3HT BCPs by the living crystallization-driven self-assembly (CDSA) method, which involves epitaxial growth of BCP unimers (molecularly dissolved BCP) from seed micelles^37^,^38^,^39^,^40^,^41^,^42^. A modification of CDSA, known as thermally induced self-seeding, was used in this study to facilitate the formation of a *rr*P3HT core with a minimized number of defects^38^,^41^,^42^. In this solution-based process, short fibre-like micelles are heated to different temperatures. Regions of lower core crystallinity dissolve to leave crystalline seeds; on cooling to room temperature, the dissolved BCP adds to the seeds to form elongated fibres. As the percentage of remaining seeds decreases with increased temperature^43^,^44^,^45^,^46^, the length of the resulting fibres can be controlled precisely to yield fibres with relatively low-length dispersities.

**Figure 1:**
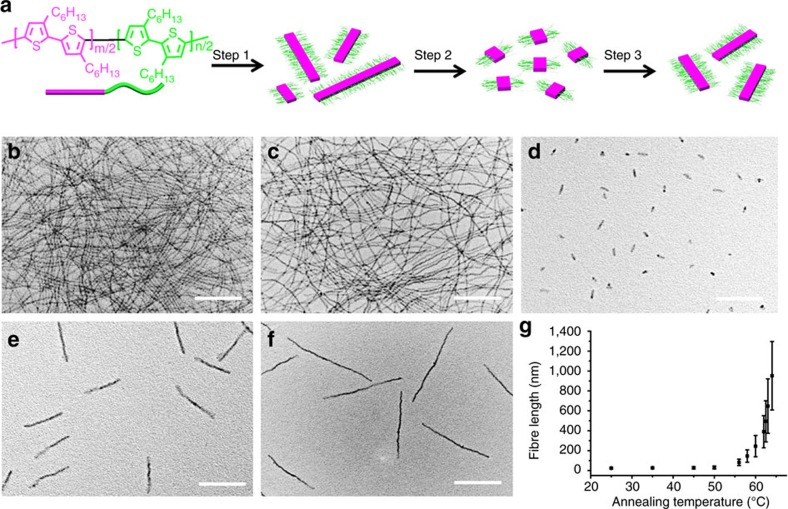
Fibres from *rr*P3HT-*b*-*rs*P3HT. (**a**) Chemical structure of *rr*P3HT-*b*-*rs*P3HT diblock copolymer and the schematic illustration of Step 1, self-assembly of *rr*P3HT-*b*-*rs*P3HT to form fibres in anisole; Step 2, fragmentation of the fibres; and Step 3, thermal annealing to form uniform fibres. TEM images of the unfragmented fibres from (**b**) *rr*P3HT_48_-*b*-*rs*P3HT_43_ and (**c**) *rr*P3HT_106_-*b*-*rs*P3HT_47,_ which were obtained by directly dispersing the polymer solid in anisole at 0.1 mg ml^−1^ at 80 °C for 1 h and then slowly cooling to 23 °C. Uniform fibres from *rr*P3HT_106_-*b*-*rs*P3HT_47_ prepared by subjecting anisole solutions of fibres (0.1 mg ml^−1^) to ultrasonication at 0 °C for 1 h, followed by thermal annealing at (**d**) 56.0 °C, (**e**) 62.5 °C, (**f**) 64.0 °C for 30 min and then slow (*ca*. 6 h) cooling to 23 °C. Scale bars, 500 nm. (**g**) Plot of the *L*_n_ of the *rr*P3HT_106_-*b*-*rs*P3HT_47_ fibres versus self-seeding temperatures.

In this study, polydisperse fibre-like micelles were initially prepared by dissolution of the BCPs in anisole at 80 °C for 1 h and subsequent cooling to room temperature over 6 h (23 °C; Fig. 1a, Step (1)). Shown in Fig. 1b,c are the transmission electron microscopy (TEM) images of the resulting multi-micrometre-long fibres, of which lengths were often larger than the TEM grid used for imaging (∼10 μm × 10 μm). The length distribution was broad and the polydispersity of the *rr*P3HT_106_-*b*-*rs*P3HT_47_ fibres (*L*_w_/*L*_n_, where *L*_w_ is the weight-average length and *L*_n_ the number average length) was ∼2.4 and ∼2.0 for *rr*P3HT_48_-*b*-*rs*P3HT_43_ fibres. Despite large differences in the DP of the *rr*P3HT block, the fibres formed from the two BCPs were found to be very similar in core width (15±2 nm and 16±1 nm as determined from TEM images for fibres from *rr*P3HT_48_-*b*-*rs*P3HT_43_ and *rr*P3HT_106_-*b*-*rs*P3HT_47_, respectively). This implies that the P3HT chains in a *rr*P3HT_48_-*b*-*rs*P3HT_43_ fibre are almost fully extended, whereas chain folding should occur in the core of *rr*P3HT_106_-*b*-*rs*P3HT_47_ fibres^47^,^48^ (see ‘Additional discussion on chain folding in the fibre core’ in the Supplementary Information). The less dense, amorphous *rs*P3HT corona was only visible after staining with RuO_4_ (Supplementary Fig. 4a). The width of the *rr*P3HT_106_-*b*-*rs*P3HT_47_ fibres increased to 26±3 nm after staining, clearly indicating the existence of an outer corona layer. The crystalline *rr*P3HT cores were characterized by photoluminescence and solution ultraviolet–visible absorbance spectroscopy, whereas *rs*P3HT is completely amorphous and soluble in anisole (see Supplementary Figs 5,6 and ‘Additional discussion on the ultraviolet–visible and photoluminescence spectra’ in the Supplementary Information). The crystalline *rr*P3HT core is lamellar and of type I polymorph, as determined by thin-film grazing incidence wide-angle X-ray scattering (GIWAXS) measurements (Supplementary Fig. 7 and ‘Additional comments on GIWAXS data’ in the Supplementary Information). Based on the results from TEM, atomic force microscopy (AFM) and GIWAXS, these fibres adopt a ribbon-like structure, with the *rs*P3HT corona chain only present on the sides of the fibres (see ‘Additional comments on GIWAXS data’ in the Supplementary Information and Supplementary Fig. 4).

The self-seeding process involved two steps. Taking the case of *rr*P3HT_106_-*b*-*rs*P3HT_47_, first, the pristine polydisperse fibre-like micelles were fragmented by ultrasonication in anisole at 0 °C for 1 h (Fig. 1a, Step (2), Supplementary Fig. 8a) to yield uniform short fibres. In the second step, the solutions of short fibres were thermally annealed at a desired temperature between 56 °C and 64 °C for 1 h, followed by gradual cooling to 23 °C over several hours (Fig. 1a, Step (3)). The maximum annealing temperature for self-seeding experiments was *ca*. 64 °C. Above this temperature, all of the short fibre-like micelles dissolved and, in the absence of remaining seeds, no length control was evident, as samples cooled to 23 °C contained mixtures of both very long and short fibres. The lengths (*L*_n_ and *L*_w_) of the resultant uniform fibres were determined from the TEM images and the s.d. (*σ*) calculated. The samples annealed at 64 °C (Fig. 1f) produced the longest fibres with a narrow length distribution (*L*_n_=952 nm, *L*_w_/*L*_n_=1.10). The length information for samples at different annealing temperatures is summarized in Fig. 1g (also Supplementary Fig. 8 and Supplementary Table 2). Based on the initial length of the short fibres and the final length of fibres after annealing, we calculated the fraction of surviving fragments at each annealing temperature. As shown in Supplementary Fig. 8j, for the investigated temperature range, the fraction of surviving seeds decreased exponentially with increasing temperature, a key characteristic of a self-seeding process^41^,^49^. This protocol thus allowed the formation of fibres ranging in length from below 30 nm to over 950 nm with low polydispersities (*L*_w_/*L*_n_<1.20). During this process, the peak intensity at 560 nm in ultraviolet–visible spectrum increased, indicating a higher crystallinity of the rrP3HT block in the core^50^ (Supplementary Fig. 6e). For *rr*P3HT_48_-*b*-*rs*P3HT_43_ fibres, the same experimental procedures were applied and similar results were obtained (Supplementary Fig. 9 and Supplementary Table 2).

### Electroactive fibre-like micelles

**Figure 2:**
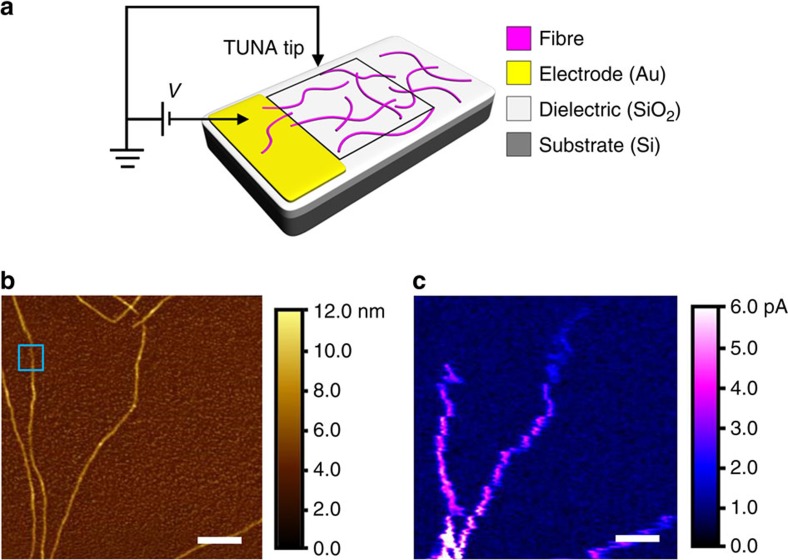
Tunnelling atomic force (TUNA) microscopy image of fibres. (**a**) Schematic of the TUNA experiment (the black square indicates where the image was obtained). (**b**) Height and (**c**) TUNA contact current images of unfragmented *rr*P3HT_106_-*b*-*rs*P3HT_47_ fibres (*L*_n_>10 μm). Scale bars, 500 nm. The fibres visible in **b** but not in **c** were disconnected from the gold electrode (break in the fibre, and thus the connection to the gold electrode, indicated in the blue box). The sub-monolayer films were prepared by spin-coating of 0.1 mg ml^−1^ fibre dispersions at 3,000 r.p.m. The gold electrode is positioned at the bottom of the AFM images and not imaged to avoid short-circuiting; see Supplementary Fig. 10a,b for an AFM height image showing the electrode position, as well as the TUNA contact current image at full scale, respectively.

The electroactive nature of the individual *rr*P3HT-*b*-*rs*P3HT fibres was demonstrated using tunnelling AFM (TUNA) experiments, as depicted in Fig. 2a. Measurements were performed using the common bottom-contact architecture, with a SiO_2_ dielectric layer (300 nm), thermally evaporated Au electrodes (30 nm) and a hexamethyldisilazane layer (deposited by spin coating). Figure 2b shows an AFM height image taken on several unfragmented *rr*P3HT_106_-*b*-*rs*P3HT_47_ fibre-like micelles (length>10 μm) in the vicinity of a thermally evaporated gold electrode on a Si/SiO_2_ substrate, as indicated by the black square in Fig. 2a. In this two-probe measurement, the Pt/Ir AFM tip and the evaporated gold were used as a pair of electrodes. The gold electrode, which is positioned at the bottom of the AFM images in Fig. 2, was not imaged to prevent a short circuit (see Supplementary Fig. 10 for the full AFM height image). The corresponding TUNA contact current image in Fig. 2c shows a current map (in pA) upon the application of a constant DC bias of 9.0 V, with the fibres clearly visible (see ‘Characterization of TUNA samples’ in the Supplementary Information). The maximum TUNA current measured was appreciable at 17.5 pA (the scale of the TUNA current image in Fig. 2c is adjusted to 6 pA, to show the image more clearly), with the tip at a distance of *ca*. 100 nm from the electrode.

### Field-effect transistors based on fibre-like micelles

**Figure 3:**
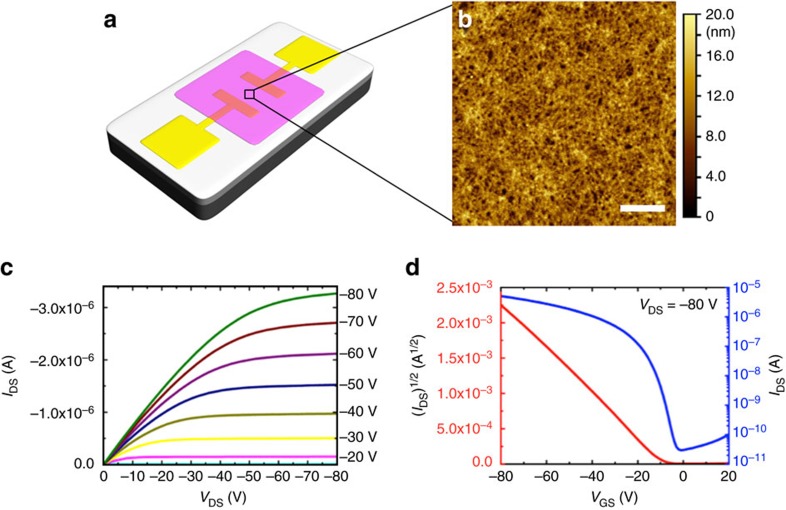
OFETs fabricated from the *rr*P3HT-*b*-*rs*P3HT and *rr*P3HT-*b*-PS fibres. (**a**) Schematic illustration of the bottom-gate, bottom-contact OFET device (structures not drawn to scale; purple: semiconducting film; yellow: gold source/drain electrodes; light grey: dielectric SiO_2_ layer; dark grey: gate Si electrode). (**b**) AFM image of *rr*P3HT_106_-*b*-*rs*P3HT_47_ fibres (cast from 0.1 mg ml^−1^ anisole solution). Scale bar, 1 μm. The representative (**c**) output and (**d**) transfer characteristics of the OFET device of the unfragmented *r*P3HT_106_-*b*-*rs*P3HT_47_ fibres (*L*_n_>10 μm) (*I*_DS_=drain-to-source current; *V*_DS_=drain-to-source bias; *V*_GS_=gate-to-source bias).

The demonstration of conductivity for these fibres, which is significantly higher than that of the substrate, encouraged us to examine thin film assemblies of the uniform fibres with different lengths (and coronas) as active layers in organic field-effect transistors (OFETs). The OFET devices were prepared on the same substrates as used for TUNA measurements (Fig. 3a). Relatively long channel lengths (*L*=30 μm or longer) were used to reduce the contribution of contact resistance to the total resistance of the devices. The channel width was kept at 1 mm for all samples unless specified.

In our initial fabricated devices, solutions (0.1 mg ml^−1^) of unfragmented fibre-like micelles (length>10 μm) prepared in anisole were deposited by spin-coating onto prepared substrates. Thin films with randomly oriented fibres (Fig. 3b and Supplementary Fig. 11) with a thickness of a few fibre layers were prepared and tested (see ‘Fabrication of GIWAXS, TUNA and OFET samples’ in the Supplementary Information for further details).

Figure 3c shows typical output and transfer characteristics obtained from OFETs fabricated from pristine, unfragmented *rr*P3HT_106_-*b*-*rs*P3HT_47_ fibres. The output curves clearly demonstrate the field effect with full channel saturation and negligible leakage. From the values of the square root of the saturation current (Fig. 3d, *V*_DS_=−80 V), we calculated the mobility *μ* to be 6.3 × 10^−3^ cm^2^ V^−1^ s^−1^ (see ‘Characterization of OFET samples’ in the Supplementary Information). Meanwhile, OFETs from fully dissolved *rr*P3HT_106_-*b*-*rs*P3HT_47_ in chloroform (that is, without the pre-organized nanofibres), showed a much lower mobility (1.9 × 10^−4^ cm^2^ V^−1^ s^−1^, see Supplementary Fig. 15).

To probe the influence of the corona on the mobility and performance of prepared OFETs, analogous studies were performed using unfragmented fibre-like micelles with a similar *rr*P3HT core and an insulating PS corona. A summary of mean field-effect mobility values (obtained over at least ten devices) for all of the materials investigated is shown in Table 1. We found that the OFETs from *rr*P3HT_54_-*b*-PS_44_ fibres (Table 1, Entry 3) exhibited very similar *μ* values as those from *rr*P3HT_48_-*b*-*rs*P3HT_43_ fibres (Table 1, Entry 2). Considering the insulating nature of PS corona chains, this finding suggests that the *rs*P3HT corona block makes a very small, if not negligible, contribution to the overall mobility in the fibres. We therefore believe that charge transport occurs through the crystalline *rr*P3HT core (that is, intra-fibre) rather than through the amorphous corona chains. In an additional control experiment, devices made from films of amorphous *rs*P3HT_90_ homopolymer showed no measurable field effect, despite their semiconductive nature, providing further support for this assertion (Supplementary Fig. 14). The apparent independence of *μ* on the corona block is also supported by the observation of similar values of *μ* obtained from *rr*P3HT_48_ homopolymer fibre-based OFET devices (Table 1, Entry 1). These similar values of *μ* from *rr*P3HT_48_-*b*-*rs*P3HT_43_ fibres, *rr*P3HT_54_-*b*-PS_44_ fibres and *rr*P3HT_48_ homopolymer fibres clearly demonstrated that charge carrier mobility is independent of corona composition. Furthermore, on increasing the DP of the *rr*P3HT block from 48 to 70, and finally to 106, *μ* also increased, regardless of the nature and length of the corona-forming block (Table 1). This increase is probably due to the higher electronic coupling along the conjugated chains and better-connected domains^51^. However, in contrast to the BCP fibres, those comprising solely *rr*P3HT homopolymer, without the steric protection of the solvated *rs*P3HT corona chains, exhibited poor colloidal stability and precipitated from anisole.

**Figure 4:**
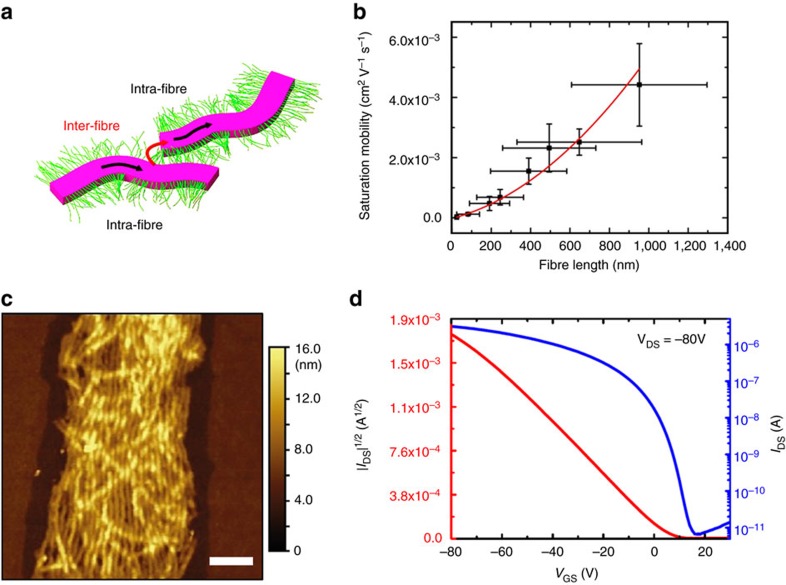
Field-effect mobility of devices from *rr*P3HT_106_-*b*-*rs*P3HT_47_ fibres. (**a**) Schematic illustration of the charge-carrier transfer processes present in thin films of semiconductive fibre networks. (**b**) Variation of saturation mobility versus the fibre lengths after self-seeding. The red curve is a quadratic fit to the data. Each saturation mobility data point is averaged over the data from at least ten devices. (**c**) AFM image of the aligned and unfragmented *rr*P3HT_106_-*b*-*rs*P3HT_47_ fibres (*L*_n_>10 μm). Scale bar, 200 nm. (**d**) Transfer characteristics of the OFET device of the aligned and unfragmented *r*P3HT_106_-*b*-*rs*P3HT_47_ fibres.

With these results clearly indicating the dependence of mobility on *rr*P3HT molar mass, we next explored the influence of overall fibre length (whilst keeping the molar mass constant). As a result of the ability to control the length of the fibres and to prepare samples with narrow length distributions through the described living CDSA self-seeding process, we prepared OFET devices with *rr*P3HT_106_-*b*-*rs*P3HT_47_ fibres of specific and predetermined dimensions. When the variation of saturation mobility was plotted against *rr*P3HT_106_-*b*-*rs*P3HT_47_ fibre length, a strong, super-linear dependence was found (Fig. 4b), with mobility values ranging over two orders of magnitude, from 2.4 × 10^−5^ to 4.4 × 10^−3^ cm^2^ V^−1^ s^−1^. A similar relationship has been reported between the charge-carrier mobility and (crystal) grain sizes for OFETs based on pentacene^52^, copper phthalocyanine,^53^ oligo(thiophene)s^54^ and P3HT crystals^55^. In all of these previously reported cases, the increase in mobility was attributed to a decreased density of grain boundaries. We therefore postulate that a process analogous to control over the density of grain boundaries takes place in our case. Here the increase in fibre length leads to an effective increase in the size of the grains. As all self-seeded fibres were randomly oriented and much shorter than the transistor channel length (30 or 50 μm), both fast intra- and slow inter-fibre charge transport have to be considered, as depicted schematically in Fig. 4a. In this case, controlling the fibre length can be equated to controlling the size of the grains in polycrystalline films of small organic molecules: longer fibres present an increase of grain size and thus an effective reduction in grain boundary density, leading to enhanced mobility. Furthermore, the observed super-linear charge-carrier mobility dependence on grain size fits well with previous models for both small molecules^56^ and thin films of crystallized P3HT^55^. Significantly, the hole mobility of uniform 950 nm fibres from *rr*P3HT_106_-*b*-*rs*P3HT_47_ (4.0 × 10^−3^ cm^2^ V^−1^ s^−1^) is higher than that from unfragmented *rr*P3HT_48_-*b*-*rs*P3HT_43_ (2.0 × 10^−3^ cm^2^ V^−1^ s^−1^). This mobility difference can once again be attributed to the molecular weight difference between the *rr*P3HT blocks. The intrachain transport contribution probably plays a far more significant role than the interchain transport, as is also supported by the mobility values obtained from unfragmented fibres (*L*_n_>10 μm): *rr*P3HT_106_-*b*-*rs*P3HT_47_ (6.3 × 10^−3^ cm^2^ V^−1^ s^−1^) and *rr*P3HT_48_-*b*-*rs*P3HT_43_ (2.0 × 10^−3^ cm^2^ V^−1^ s^−1^), respectively.

The mobility values obtained (*μ*∼10^−3^ cm^2^ V^−1^ s^−1^) are comparable with those for early P3HT field-effect devices^57^,^58^, but are significantly lower than more recent and optimized systems^59^ (*μ*∼10^−1^ cm^2^ V^−1^ s^−1^). In general, improvements to overall device performance would be anticipated through similar optimization strategies that involve the use of high molar mass P3HT^48^,^60^, higher boiling point solvents^61^ or thin-film annealing procedures^48^,^51^. Furthermore, as it has been demonstrated that the alignment of carbon nanotubes^62^ and semiconductive polymer chains substantially enhances charge-carrier mobility^27^,^63^, alignment of the BCP fibres should also lead to increased mobilities.

Our preliminary results indicate this to be the case and unoptimized mobility values of over 1.5±0.16 × 10^−2^ cm^2^ V^−1^ s^−1^ were achievable using alignment via a dip-coating procedure. An AFM image of the aligned unfragmented *r*P3HT_106_-*b*-*rs*P3HT_47_ fibres is shown in Fig. 4c and the transfer curves from the corresponding FET device in Fig. 4d. With this dip-coating procedure, most of the micelles were aligned between the two electrodes (for experimental details and more characterization of the sample, see ‘Preparation and testing of OFET with aligned fibres’ in the Supplementary Information and Supplementary Fig. 13). Clearly, this alignment greatly enhanced the charge-carrier mobility (which increased from 6.3 × 10^−3^ to 1.5 × 10^−2^ cm^2^ V^−1^ s^−1^). In addition to the wide range of alignment conditions available, the control over fibre length, corona type and, finally, type of semiconducting core material, provides a myriad of potential opportunities for further improvement in device performance. The mobility enhancement after alignment (*ca*. 140%) is encouraging and further investigations to improve hole mobility are ongoing and will be reported in the near future.

## Discussion

In summary, fibre-like micelles with a well-defined core–corona structure prepared from a π-conjugated BCP have been shown to possess promising electroactive properties in single-fibre TUNA–AFM investigations. Their preparation by the living CDSA self-seeding method yields low dispersity samples with finely controlled lengths and excellent colloidal stability provided by the corona chains, which has allowed a detailed evaluation of their performance in bottom-contact field-effect transistors. The charge-carrier mobilities are independent of corona composition, increase with molar mass of the electroactive core-forming block and, more importantly, also increase in a super-linear manner with fibre length. Although the mobility values in this proof-of-concept work were relatively modest, obvious future avenues exist for the improvement of device performance as shown by the values obtained from aligned-fibre devices. Moreover, as the versatile living CDSA method should be applicable to other crystallizable π-conjugated semiconducting polymers (and other corona blocks), opportunities exist for the incorporation of a wide range of uniform electroactive micelle-based nanostructures into devices, including more complex architectures such as segmented structures^38^,^64^ or micelle-nanoparticle hybrids^12^,^13^,^65^,^66^.

## Methods

### Materials

Methanol (MeOH), hexane, anisole, 1,2-dichlorobenzene, 2,5-dibromo-3-hexylthiophene, chloroform, lithium chloride (LiCl), [1,3-bis(diphenylphosphino)propane]dichloronickel(II) (Ni(dppp)Cl_2_) and *tert*-butylmagnesium chloride (1.0 M in tetrahydrofuran (THF)) were purchased from Sigma-Aldrich and used as received. 5,5′-Dibromo-3,3′-dihexyl-2,2′-bithiophene was purchased from Tokyo Chemical Industry and used as received. Cyclohexane and THF were purchased from Fisher Scientific. THF for polymerizations was distilled over sodium and benzophenone before use. Hexamethyldisilazane (electronic grade) was purchased from Alfa Aesar and was used as received. Octadecyltrichlorosilane was purchased from Acros Organics.

### Synthesis of *rr*P3HT-*b*-*rs*P3HT

The diblock copolymers were synthesized via sequential polymerization of the two monomers, 2,5-dibromo-3-hexylthiophene and 5,5′-dibromo-3,3′-dihexyl-2,2′-bithiophene via the GRIM method^67^,^68^,^69^. For a typical synthesis route for *rrP*3HT_48_-*b*-*rs*P3HT_43_, 2,5-dibromo-3-hexylthiophene (324 mg, 1 mmol) (solution 1) and 5,5′-dibromo-3,3′-dihexyl -2,2′-bithiophene (246 mg, 0.5 mmol) (solution 2) were dispersed into 5 ml of THF in two separate vials. *tert*-Butylmagnesium bromide solution (1 eq. mol to the monomer) was added dropwise into the two solutions. Both solutions were stirred for 2 h in the dark. Another 20 ml of THF was added into solution 1 before 10.8 mg (0.02 mmol, 2% mol) of Ni(dppp)Cl_2_ was added in one portion. After 30 min of stirring, solution 2 was added into solution 1 and the resultant mixture was allowed to stir overnight. The reaction was quenched by adding HCl aqueous solution (5%). (All the reactions, except the quenching, were conducted at room temperature (23 °C) and in a glove box under Ar atmosphere (O_2_ and H_2_O level<2 p.p.m.).) Chloroform (100 ml) and H_2_O (50 ml) were added into the solution and the polymer was extracted into organic layer. The organic layer was separated and dried under vacuum. The solid was then purified by Soxhlet extraction, using MeOH and hexane as solvent. The purified polymer was collected by dissolving in chloroform, concentrating under vacuum, precipitating into MeOH and obtained by centrifugation. The diblock copolymers were further fractionated by passing through a size exclusion chromatography column with THF as eluent. A soft dark purple solid (103 mg) was obtained with a yield of 18%. The synthesis of *rr*P3HT-*b*-PS has been described elsewhere^38^ and is not repeated here. The Cu salt was removed by passing through basic alumina and silica gel columns repeatedly. Based on the elemental analysis results (graphite furnace atomic absorption), the content of Cu content is very low, at the p.p.m. level, and would not influence the FET device performance significantly^70^. For characterization data, see Supplementary Table 1.

### Polymer characterization

Gel permeation chromatography was carried out on a Viscotek VE 2001 Triple-Detector Gel Permeation Chromatograph equipped with an automatic sampler, a pump, an injector, an inline degasser and a column oven (30 °C). The elution columns consist of styrene/divinyl benzene gels with pore sizes of 500 Å and 100,000 Å. Detection was conducted by means of a VE 3580 refractometer, a four-capillary differential viscometer, and 90^o^ and low angle (7^o^) laser light (*λ*_0_=670 nm) scattering detectors, VE 3210 and VE 270. THF was used as the eluent, with a flow rate of 1.0 ml min^−1^. Samples were dissolved in THF (2 mg ml^−1^) and filtered with a Ministart SRP 15 filter (polytetrafluoroethylene membrane of 0.45 μm pore size) before analysis. The calibration was conducted using a PolyCALPS standard from Viscotek. To determine the molecular weight of the BCPs, aliquots of the first block were taken and the absolute molecular weight of the first block was determined by matrix-assisted laser desorption/ionization–time of flight mass spectrometry. The absolute polymerization degrees of the two blocks were then determined by combining the molecular weight *M*_n_ of the first block from matrix-assisted laser desorption/ionization–time of flight mass spectrometry measurements with the block ratio of the diblock copolymer obtained by integration of the ^1^H NMR spectrum. The regioregularities of the polymers were determined from their ^1^H NMR spectra. The differential scanning calorimetry and thermogravimetric analyses were performed on the Q100 and Q500 from TA instruments at heating/cooling rates of 10 °C min^−1^. The differential scanning calorimeter was coupled to a refrigerated cooling system (RCS90).

### Self-assembly of *rr*P3HT-*b*-*rs*P3HT in solvents

Typically, the diblock copolymers were directly dispersed into anisole at a concentration of 1 mg ml^−1^ in tightly sealed vials. The solution was heated at 80 °C for 2 h without stirring before cooling down to room temperature. The solutions were bright orange at high temperatures and turned purple in several hours after cooling to room temperature. The *rr*P3HT-*b*-PS fibres were prepared by exactly the same method. Fibres from *rr*P3HT_48_ homopolymer were prepared in the same way as the diblock copolymers, except that the fibres were not quite colloidally stable and a precipitate was formed after storage at room temperature for several days. All the pristine fibres formed by the diblock copolymers and homopolymer were longer than 10 μm and were polydisperse in length.

### Preparation of fibres with controlled length via the self-seeding method

Fibres prepared in anisole (after 2 days of ageing at 23 °C) were subjected to sonication in a sonication bath (160 W) at 0 °C for 60 min to yield seed fibres. The seed solution was heated at different temperatures for 30 min then cooled and aged at room temperature to allow the nanofibres to grow.

It is worth noting that the self-seeding window for *rr*P3HT_106_-*b*-*rs*P3HT_47_ fibres is much higher and narrower than that for the *rr*P3HT_48_-*b*-*rs*P3HT_43_ fibres. This can be better appreciated by plotting the length of the fibres versus annealing temperatures, as shown in Fig. 1g and Supplementary Fig. 9f. We believe that this difference can be attributed to the much higher DP of the *rr*P3HT block for *rr*P3HT_106_-*b*-*rs*P3HT_47_, resulting in higher crystallinity of the fibres. This was supported by the more significant red-shift of the ultraviolet–visible absorbance spectra of the nanofibre solution from *rr*P3HT_106_-*b*-*rs*P3HT_47_, as shown in Supplementary Fig. 6.

### Data availability

All data are available from the authors upon reasonable request.

## Additional information

**How to cite this article:** Li, X. *et al*. Uniform electroactive fibre-like micelle nanowires for organic electronics. *Nat. Commun.* **8**, 15909 doi: 10.1038/ncomms15909 (2017).

**Publisher’s note:** Springer Nature remains neutral with regard to jurisdictional claims in published maps and institutional affiliations.

## Supplementary Material

Supplementary Information

## Figures and Tables

**Table 1:** Mean field-effect mobility and on/off ratios obtained from at least ten devices for all unfragmented (length>10 μm) fibres investigated in this work.

**Fibre type**	**Mobility (× 10**^**−3**^ **cm**^**2**^ **V**^**−1**^ **s**^**−1**^**)**	**On/off ratio**
*rr*P3HT_48_	2.1±0.3	5 × 10^4^
*rr*P3HT_48_-*b*-*rs*P3HT_43_	2.0±0.6	5 × 10^5^
*rr*P3HT_54_-*b*-PS_44_	1.7±0.2	2 × 10^5^
*rr*P3HT_70_-*b*-PS_197_	3.4±0.1	8 × 10^4^
*rr*P3HT_106_-*b*-*rs*P3HT_47_	6.3±1.0	2 × 10^5^
