# Erratum: Uniform electroactive fibre-like micelle nanowires for organic electronics

**DOI:** 10.1038/ncomms16142

**Published:** 2017-07-27

**Authors:** Xiaoyu Li, Piotr J. Wolanin, Liam R. MacFarlane, Robert L. Harniman, Jieshu Qian, Oliver E. C. Gould, Thomas G. Dane, John Rudin, Martin J. Cryan, Thomas Schmaltz, Holger Frauenrath, Mitchell A. Winnik, Charl F. J. Faul, Ian Manners

Nature Communications 8 Article number: 15909 ; DOI: 10.1038/ncomms15909 (2017); Published 06 26 2017; Updated 07 27 2017.

An incorrect version of the Supplementary Information was inadvertently published with this Article where the wrong file was included. The Article has been updated to include the correct version of the Supplementary Information.

