# Supplementary material for: Uniform electroactive fibre-like micelle nanowires for organic electronics
Source: Nat Commun. 2017 Jun 26;8:15909. doi: 10.1038/ncomms15909 (PMC5490183; doi:10.1038/ncomms15909)
Supplement: Supplementary Information [file ncomms15909-s1.pdf]

Supplementary Methods

Transmission electron microscopy (TEM). Samples were prepared by drop-casting one drop (ca. 10 μL) of the solution onto a carbon-coated copper grid which was placed on a piece of filter paper to remove excess solvent. Bright field TEM images were obtained on a JEOL1200EX II microscope operating at 120 kV and equipped with an SIS MegaViewIII digital camera. No staining of the samples was necessary. Staining of the samples with RuO_4 was carried out by exposing the sample grid to RuO_4 vapor for overnight in a covered Petri dish.

Nanofiber length distributions were determined using the software program ImageJ from the U.S. National Institutes of Health. For each sample, ca. 100 fibers from several images were traced by hand in order to obtain the length information. The number average nanofiber length (L_n) and weight average nanofiber length (L_w) were calculated using Supplementary Equation 1 from measurements of the contour lengths (L_i) of individual fibers, where N_i is the number of fibers of length L_i, and n is the number of fibers examined in each sample.

$$L_n = \frac{\sum_{i=1}^{n} N_i L_i}{\sum_{i=1}^{n} N_i} \qquad L_w = \frac{\sum_{i=1}^{n} N_i L_i^2}{\sum_{i=1}^{n} N_i L_i} \qquad (1)$$

The distribution of nanofiber lengths is characterized by both L_w/L_n and the ratio σ/L_n, where σ is the standard deviation of the length distribution.

Grazing-incidence wide-angle X-ray scattering (GIWAXS). GIWAXS measurements were performed at beamline ID13, the microfocus beamline at ESRF – the European Synchrotron (Grenoble, France). Monochromatic X-rays (energy $E = 13$ keV, wavelength $\lambda = 0.9537$ Å) were focused to a spot size of $2 \times 2\ \mu m^2$ by beryllium compound refractive lenses. Diffraction patterns were collected on an EIGER 4M detector (DECTRIS) positioned 230.55 mm from the sample. The detector geometry was calibrated by aluminium oxide powder in transmission mode using the *pyFAI-calib* command line tool[1]. Samples were aligned to the focal plane of the X-ray beam by use of a calibrated optical microscope. A fixed X-ray incident angle of 0.2° was used, which gave a footprint on the sample surface of ~$500 \times 2\ \mu m^2$. The samples were scanned normally to the beam and the resulting diffractions patterns were averaged to improve the data statistics. The data were projected into reciprocal space coordinates and line profiles were extracted using the python library, *pygix*[2]. Out-of-plane line profiles were extracted by integration of a sector of data along q_z with an angular width of 25°. In-plane line were extracted by integration of box of data along q_{xy} at fixed $q_z = 0.5\ nm^{-1}$, with a width of $\Delta q_z = 0.5\ nm^{-1}$. These integration regions are shown in Supplementary Fig. 7a.

Fabrication of GIWAXS, TUNA and OFET samples. All samples were prepared on highly doped silicon substrates having a nominal thickness of 300 nm of thermally grown SiO_2, which were obtained from Ossila Ltd. Prior to use, they were cleaned by ultrasonication for 5 minutes in, sequentially, acetone and *iso*-propanol, then rinsed with deionized water, and dried with a nitrogen gun. This was followed by

spin-coating of hexamethyldisilazane at 2000 RPM for 30 s in order to remove the –OH groups from the surface.

In the case of TUNA and OFET devices, source/drain gold electrodes (30 nm) were thermally evaporated onto bare substrates through a shadow mask (Ossila Ltd.). Electrode deposition took place in a class 10000 cleanroom using an Edwards evaporator at a starting pressure of < 3.0 x 10^{-6} mbar and evaporation rate of < 1 Å s^{-1}. Relatively long channel lengths (L = 30 µm to L = 80 µm) were used in order to reduce the contribution of contact resistance to the total resistance of the devices; unless stated, the channel width was kept constant at 1 mm. Due to a relatively low mobility of the *rr*$P3HT_{106}$-*b*-*rs*$P3HT_{47}$ seeds, a mask with a higher W/L ratio (1823 µm / 50 µm) was used to allow an appreciable amount of current through the devices.

Fiber dispersions (0.1 mg mL^{-1} in anisole) were deposited by spin-coating under N_2 in a glovebox. OFET samples were spun using a two-step program: first at 100 rpm for approximately 2 h to evaporate most of the solvent and then at 1500 rpm for 90 s to produce uniform, high-quality thin-films with a thickness of at least 10 nm. OFETs for *rs*$P3HT_{90}$ were fabricated the same manner except that the polymer was dissolved in anisole (5.0 mg mL^{-1}). To fabricate OFETs from *rr*$P3HT_{106}$-*b*-*rs*$P3HT_{47}$ chloroform solution, a solution of a concentration of 1.0 mg mL^{-1} and a spin rate of 1500 rpm were used.

For GIWAXS samples, step 2 was omitted in order to produce thicker films (up to ~100 nm) and thus obtain higher signal-to-noise ratio from X-ray studies. On the other hand, TUNA samples were spun at 3000 rpm for 90 s and resulted in sparsely lying fibers on the surface with occasional, denser networks; after spin-coating, each gold electrode was shortened with the highly-doped silicon substrate using a conducting silver paste and mounted onto an AFM stub.

Characterization of TUNA samples. Initial topographic investigation of samples was conducted using both a Multi-mode VIII with Nanoscope V controller utilising PeakForce feedback control and a Dimension 3100 in intermittent-contact mode (Bruker, CA, USA). In the case of the Multi-mode system Bruker SCANASYST-AIR-HR cantilevers (nominal tip radius ~ 2 nm, nominal stiffness 0.4 N m^{-1}) were used, in the case of the Dimension 3100 SCOUT cantilevers (nominal tip radius 7 nm, nominal spring constant 42 N m^{-1}) were used (NuNano, Bristol, UK).

TUNA experiment was conducted upon the multi-mode system, initially using an additional fast-scan module to generate a large-area (40 µm x 10 µm) topographic map of a region adjacent to an electrode, and then with a PF-TUNA module (noise 50 fA). The position of the probe over the area of interest was recorded via an optical alignment camera to enable accurate positioning of the PFTUNA cantilever (Pt/IR coated, nominal tip radius 20 nm) after initial high-resolution topographic mapping. A region (4.8 µm x 4.8 µm) within the mapped area and close to the electrode was then investigated via PeakForce TUNA with an applied sample bias of 9.0 V and a pre-amplifier sensitivity of 20 pA V^{-1}. In this way, the current flowing from the gold electrode, along the length of a micelle to the cantilever was measured and recorded per pixel as an image with the tip scanning toward the electrode. The measured current showed a defined trend, increasing as the lateral separation of the tip and

electrode, and thus the length of micelle in the circuit, decreased. Localized variations in the conductivity in small regions along the length of the fiber are observed. The correlation between these regions and small height variations as the micelles undulate over nanoscale surface features implies that the variation in current is due to minute changes in conductive tip interaction area. There is also a small possibility that, scanning at a constant tip-sample interaction force below 1 nN, changes might be due to small regional variations in the stiffness of the micelles.

Characterization of OFET samples. Before the measurement, each OFET sample was first stored under vacuum for at least 12 h. The current-voltage characteristics were measured under N_2 and in the dark using an Agilent 4155B semiconductor parameter analyser. A custom-written LabVIEW virtual instrument was used for controlling the equipment. The field-effect mobility (μ) was calculated from the saturation regime (drain-source voltage V_{DS} = -80 V) by deriving mobility from the MOSFET model equation[3] for drain-source current: $I_{DS} = \mu CW/2L\ (V_{GS} - V_{TH})^2$. The mobility was then obtained by fitting a straight line to the square root of the I_{DS} data and averaged over a set of 10-20 devices per each type of material investigated[4]. The mobility for all materials was found to be roughly constant for L ranging from 30 μm to 80 μm and W of 1 mm (not shown). Care was taken not to overestimate the mobility[5].

Preparation and testing of OFET with aligned fibers. We also prepared OFET devices with patterned and aligned unfragmented *rr*P3HT$_{106}$-*b*-*rs*P3HT$_{47}$ fibers (L_n > 10 μm). That was carried out by depositing them onto Si/SiO_2 substrates that were alternately functionalised with HMDS and OTS.

Substrate patterning was performed by means of photolithography using a Karl Suss MJB-3 mask aligner and a Microchem ma-N 2403 negative photoresist. After exposure and development of the resist, the substrates were immersed into 10 mM solution of OTS in HPLC grade cyclohexane overnight. After OTS monolayer formation, ma-N 2403 was removed and HMDS was deposited by spin-coating onto the previously masked regions. The HMDS regions were narrow lines (0.6 μm wide, mm-long), easily wetted by the fibers dispersed in anisole (0.04 mg mL^{-1}), whereas the OTS regions were wider (2.4 μm wide, mm-long) and displayed very poor wettability (Supplementary Fig. 13a).

The fibers were deposited by dip-coating using a custom-built setup, consisting of a small stepper motor powered from an Arduino board and controlled via computer interface. The speed at which the substrates were pulled from fiber dispersions was approximately 4 mm min^{-1}. The dip-coating procedure was carried out under ambient atmosphere. Since, the areas functionalized with HMDS were narrower than the length of the fibers, their alignment could be easily achieved (Supplementary Fig. 13c).

After deposition, 30 nm of gold were evaporated to produce top-contact OFETs (Supplementary Fig. 13b). Before electronic testing in a glovebox filled with N_2, the samples were first stored under vacuum for at least 12 h. For calculating device mobility, the channel width W was measured by using the mean width of the areas containing fibers, which was obtained from optical images taken inside the channels

of the devices. Example I-V curves are shown in Figure 4d and Supplementary Fig. 13c. The mean mobility obtained from aligned *rr*$P3HT_{106}$-*b*-*rs*$P3HT_{47}$ fibers was 0.015 ± 0.0016 cm^2 $V^{-1}s^{-1}$. We also fabricated top-contact OFETs from unfragmented *rr*$P3HT_{106}$-*b*-*rs*$P3HT_{47}$ fibers via spin-coating (unaligned fibers). These devices show a much lower average mobility of $3.2 \times 10^{-3} \pm 0.9 \times 10^{-3}$ cm^2 $V^{-1}s^{-1}$, suggesting that alignment of these fibers led to greatly enhanced mobility.

Other techniques. All the ^1H NMR characterizations were carried out on a Varian 500 MHz instrument, with chemical shifts referenced to tetramethylsilane (TMS) in *d*-chloroform ($CDCl_3$). UV-vis data were acquired from a Lambda 35 spectrometer using standard quartz cells from wavelength of 300 nm to 700 nm. Fluorescence data were obtained from a Perkin Elmer LS 45 Fluorescence Spectrometer.

Supplementary Discussions

Additional comments on polymerization sequence. Significantly, for the synthesis of diblock copolymers containing *rr*P3HT and *rs*P3HT the sequence of monomer addition cannot be reversed. In a control experiment, we attempted to synthesize *rs*P3HT-*b*-*rr*P3HT via the same GRIM method but with reversed monomer addition sequence. However, even though *rs*P3HT homopolymer could be successfully obtained, the Ni catalyst could not successfully transfer from *rs*P3HT onto 2,5-dibromo-3-hexylthiophene monomers. Instead, the newly added monomers self-polymerized, forming a new *rr*P3HT homopolymer. A plausible explanation for this is that the weakly associated π-complex of *rs*P3HT chain and Ni (0) was not as stable as that of 2-bromo-5-chloromagnesium-3-hexylthiophene monomer (*rr*P3HT monomers) and Ni (0)[6], so the Ni (0) was removed from the *rs*P3HT chain to initiate the polymerization of *rr*P3HT monomers.

Additional discussion on the self-seeding process. In Fig. 1g in the main text, we present a plot showing the average contour length vs. annealing temperature. This data illustrates that the length of the fibers increases with the annealing temperature, giving fibers up to values of ca. 950 nm with a narrow length distribution ($L_w / L_n < 1.2$).

Since the mass of polymer m in solution is constant, the length of fibers L_n is related to the total number of fibers N by Supplementary Equation 2, if we assume i) the concentration of free polymer chains at 23 °C is negligible, ii) the mass of polymer per unit length M_L remains constant.

$$L_n = \frac{m}{M_L N} \tag{2}$$

Although we could not rule out the possibility of the presence of unimers in the nanofiber solution, we checked the nanofiber solutions that were subjected to the annealing treatment 6 months after sample preparation. We found the length of the fibers did not show a significant increase, suggesting that the amount of unimers in solution at 23 °C was negligible.

Based on Supplementary Equation 2, we could calculate the fraction of seeds that survived the annealing at each temperature by taking into account the initial length of the fragments and the final length after annealing. The results are summarized in Supplementary Fig. 8j and 9g.

Additional discussion on chain folding in the fiber core. The critical molecular weight for *rr*P3HT below which the polymer chains should be fully extended is about 10 kDa (~60 repeat units)[7]. Assuming a width of 0.387 nm for a single unit of *rr*P3HT, that gives the width of a fully extended *rr*P3HT_{48} chain to be ~19 nm, which is very similar to *rr*P3HT_{48}-*b*-*rs*P3HT_{43} fiber width as measured via TEM without staining (15 ± 2 nm). On the other hand, a fully extended chain of *rr*P3HT_{106} would have to be 41 nm long, however we measured a mean width of only 16 ± 1 nm. Therefore we presume that a single *rr*P3HT_{106} chain folds. This is consistent with the minimum nanofiber height measured using AFM, as shown in Supplementary Fig. 4b, c, where the minimum nanofiber thickness is about is ~3.2 nm.

Additional discussion on the UV-vis and PL spectra. The UV-vis and photoluminescence (PL) properties of the diblock copolymer unimers and fibers were characterized and the spectra are shown in Supplementary Fig. 6. Both *rr*$P3HT_{48}$-*b*-*rs*$P3HT_{43}$ and *rr*$P3HT_{106}$-*b*-*rs*$P3HT_{47}$ polymers show a single, broad peak around 400 nm for UV-vis spectra when dissolved in chloroform as unimers. Such wavelength is much lower than *rr*P3HT homopolymers and other P3HT-containg diblock copolymers, since it is comprised of the peaks from both *rs*P3HT and *rr*P3HT block[8]. Compared to *rr*P3HT block, the *rs*P3HT block has a larger bandgap, leading to an absorbance peak at lower wavelength. Moreover, *rs*P3HT shows identical UV-vis spectra in chloroform, anisole and even in solid state, suggesting that this polymer is highly amorphous (Supplementary Fig. 6f). When the solvent was changed from chloroform to anisole, in the *rr*$P3HT_{48}$-*b*-*rs*$P3HT_{43}$ sample, shoulder peaks around 550 and 600 nm appeared, indicating the aggregation and π-π stacking of the *rr*P3HT block after nanofiber formation (Supplementary Fig. 6a). For *rr*$P3HT_{106}$-*b*-*rs*$P3HT_{47}$, a much more significant red shift of the UV spectrum could be observed (Supplementary Fig. 6b), resulting from the much longer *rr*P3HT block. The PL intensity of both polymer solutions decreased greatly, indicating the aggregation and self-quenching of the *rr*P3HT block in the core of the fiber-like micelles (Supplementary Fig. 6c, d). Photographs of these solutions under room light and UV light were included in Supplementary Fig. 5.

Additional comments on GIWAXS data. The GIWAXS data obtained from *rr*$P3HT_{48}$-*b*-*rs*$P3HT_{43}$ and *rr*$P3HT_{106}$-*b*-*rs*$P3HT_{47}$ confirmed the crystalline structure of the regioregular core (Supplementary Fig. 7). The polymer forms primarily type I polymorph with a lamellar spacing of about 1.6 nm, non-interdigitated hexyl chains and π-π stacking distance of 0.38 nm.

However, there seems to be a small amount of type II polymorph present as well, which has a smaller lamellar spacing and thus interdigitated side chains. This was inferred from the peaks for *rr*$P3HT_{106}$-*b*-*rs*$P3HT_{47}$ fibers at 1.7 nm^{-1}, 5.2 nm^{-1} and 6.9 nm^{-1} in Supplementary Fig. 7b and the peak at 12.6 nm^{-1} in Supplementary Fig. 7c (highlighted in blue circles). The former correspond to a superlattice structure formed by polymorph II[9], whereas the latter indicates a relatively large π-π stacking distance of 0.49 nm.

The reason for the presence of type II polymorph is not entirely clear, but we speculate it might have formed from remaining free polymer during the preparation of the films[9] or defects in the *rr*P3HT core. Regardless of the polymorph, *rr*P3HT adopts an edge-on orientation with respect to the substrate surface with π-π stacking along the nanofiber length.

Additionally, from AFM images, such as the example in Supplementary Fig. 4b and its corresponding line profiles in Supplementary Fig. 4c, we deduced that the nanofiber core is composed of 2-5 layers of P3HT and therefore propose a ribbon-like structure with the *rs*P3HT corona present only on the sides of the fiber; these free corona polymer chains are able to arrange and lie on the substrate surrounding the core structure. See Supplementary Fig. 4d for a schematic representation of this arrangement.

Estimation of area fill factor in OFET devices. The amount of empty space in a thin-film composed of networks of fibers can be analysed by using simple AFM image processing. AFM images are generally saved as bitmaps that are composed of pixels and each of them has a colour defined in the RGB (red, green, blue) range. Therefore, a threshold colour for the unfilled areas can be arbitrarily defined using the eyedropper tool in software such as Adobe Photoshop or MS Paint. Pixels with an RGB value below the threshold will be then considered "empty" and can be counted by iterating through all the pixels in the picture.

Shown in Supplementary Fig. 12b is an example AFM image of *rr*P3HT$_{106}$-*b*-*rs*P3HT$_{47}$ fibers grown at 64.0 ^{o}C. The corresponding analysed equivalent is shown in Supplementary Fig. 12c, where all the pixels with R < 53, G < 33 and B < 43 values have been counted and changed to white colour (R = 255, G = 255, B = 255) for visual inspection. Then, knowing the number of empty pixels enables the calculation of the fill factor, where in the case of Supplementary Fig. 12c it was 90 %:

$$\text{Area fill factor} = 1 - \frac{\text{number of empty pixels}}{\text{total number of pixels}} \times 100\% \quad (3)$$

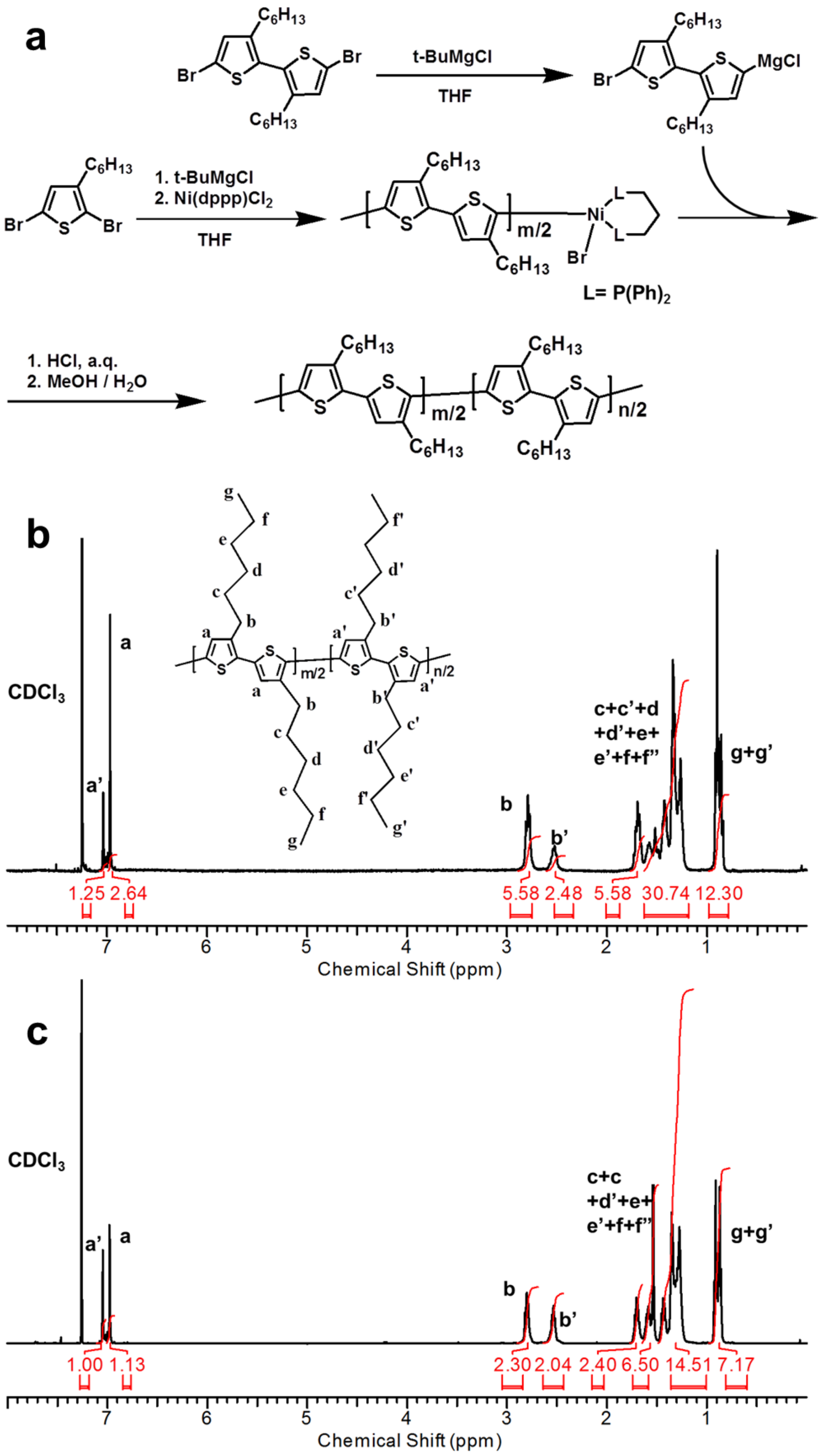

Supplementary Figure 1. (a) Schematic synthesis route for *rr*P3HT-*b*-*rs*P3HT diblock copolymers via the GRIM method. ^{1}H NMR spectra of the **(b)** *rr*$P3HT_{106}$-*b*-*rs*$P3HT_{47}$ diblock copolymer; and **(c)** *rr*$P3HT_{48}$-*b*-*rs*$P3HT_{43}$ diblock copolymer in $CDCl_3$.

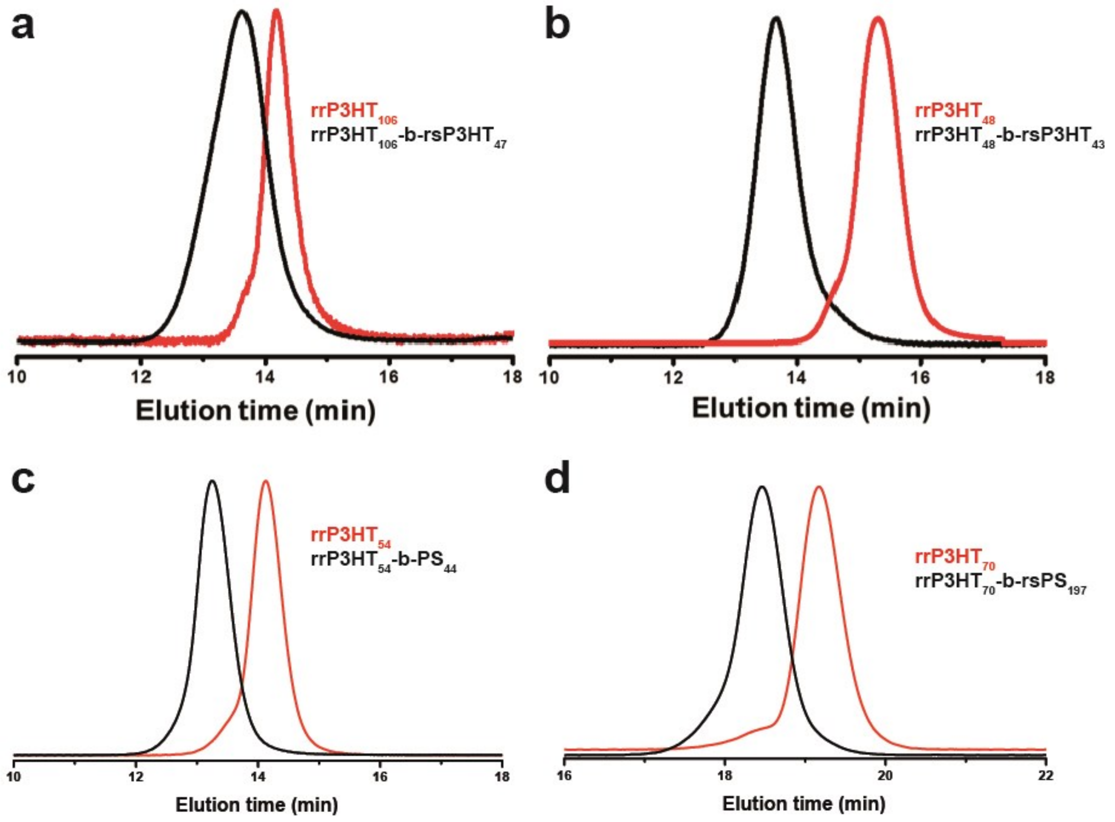

Supplementary Figure 2. GPC traces of the (a) *rr*P3HT$_{106}$-*b*-*rs*P3HT$_{47}$, (b) *rr*P3HT$_{48}$-*b*-*rs*P3HT$_{43}$, (c) *rr*P3HT$_{54}$-*b*-PS$_{44}$, (d) *rr*P3HT$_{70}$-*b*-PS$_{197}$ and their corresponding *rr*P3HT homopolymers. THF was used as eluent.

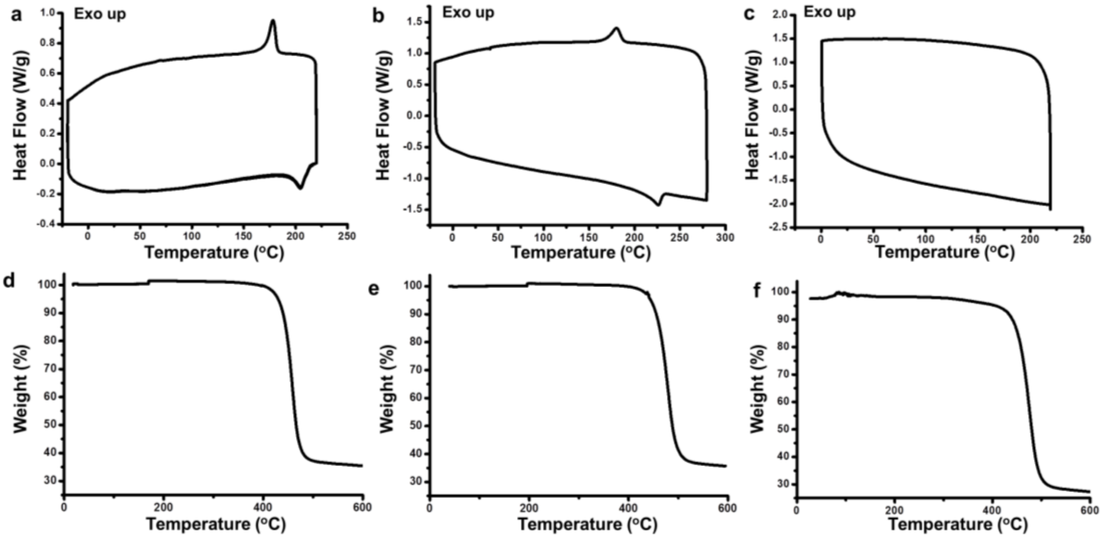

Supplementary Figure 3. DSC (a, b, c) and TGA (d, e, f) traces of the diblock copolymer *rr*P3HT$_{48}$-*b*-*rs*P3HT$_{43}$ (a, d), *rr*P3HT$_{106}$-*b*-*rs*P3HT$_{47}$ (b, e) and *rs*P3HT$_{90}$ homopolymer (c, f). DSC shows a melt transition, T_m, at 203 °C and 225 °C, respectively, as well as crystallization exotherms on the return scan. The scan rate was 10 $^oC\ min^{-1}$ in all the cases.

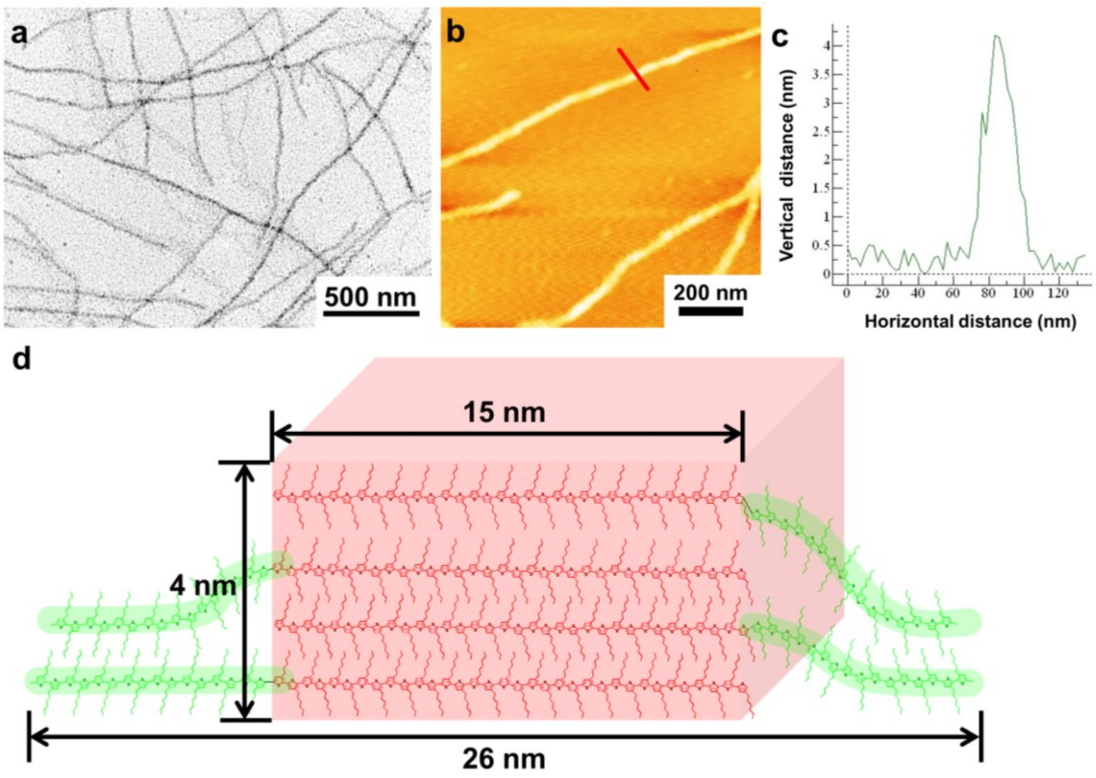

Supplementary Figure 4. (a) TEM image, (b) AFM image, (c) AFM height profile and (d) a schematic cartoon of the chain packing geometry (not drawn to scale) of a *rr*$P3HT_{106}$-*b*-*rs*$P3HT_{47}$ fiber. The sample shown in image (a) was stained with RuO_4. The *rs*P3HT corona is clearly visible on thicker fibers shown in (b).

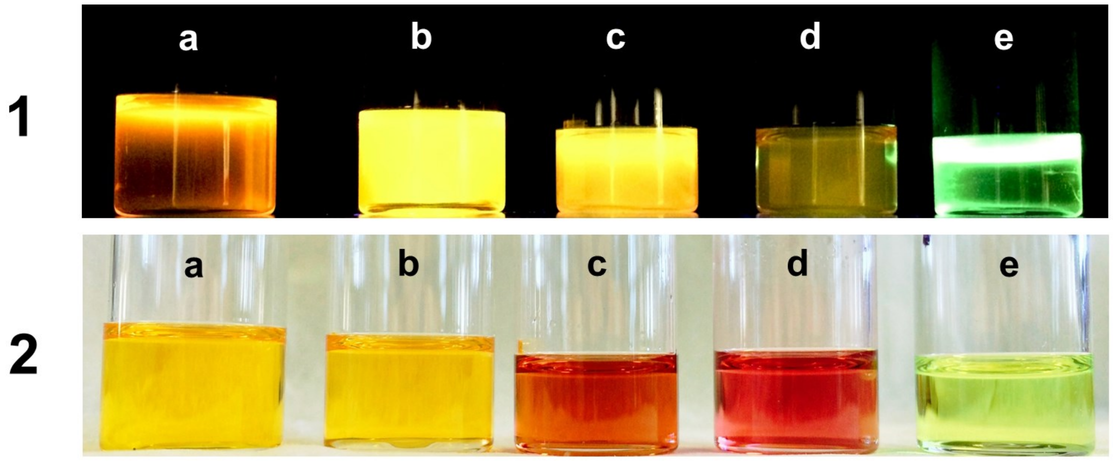

Supplementary Figure 5. Photographs of *rr*$P3HT_{48}$-*b*-*rs*$P3HT_{43}$ in (a) chloroform and (c) anisole, *rr*$P3HT_{106}$-*b*-*rs*$P3HT_{47}$ in (b) chloroform and (d) anisole, and (e) *rs*$P3HT_{90}$ in chloroform at 0.02 mg mL^{-1}. Photographs in row 1 were taken under UV light (λ = 365 nm) and those in row 2 were taken under ambient light.

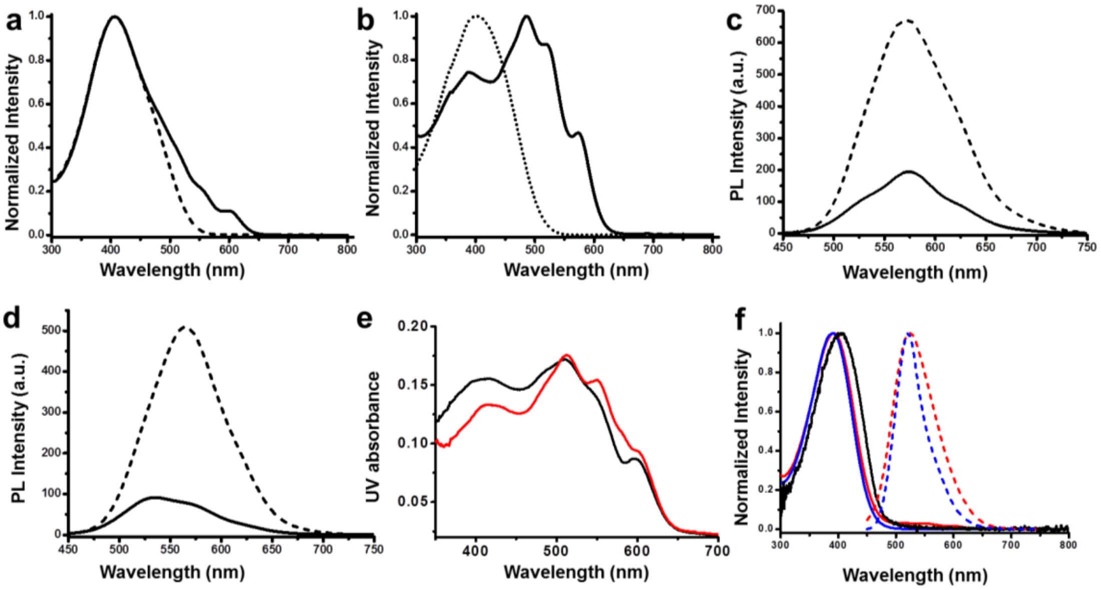

Supplementary Figure 6. (a, b) UV-vis spectra and (c, d) PL spectra of the (a, c) *rr*P3HT$_{48}$-*b*-*rs*P3HT$_{43}$ and (b, d) *rr*P3HT$_{106}$-*b*-*rs*P3HT$_{47}$ unimers in chloroform (dashed line) and fibers in anisole (solid line) (for PL spectra, λ_{ex} = 400 nm). (e) UV-vis spectra of the anisole solution of *rr*P3HT$_{106}$-*b*-*rs*P3HT$_{47}$ seeds (black), and fibers obtained by thermal annealing at 64.0 ^{o}C (red). (f) UV-vis (solid line) and PL (dashed line) spectra of anisole solution (red), chloroform solution (blue), and thin film (black) of *rs*P3HT$_{90}$ (The spectra were normalized in intensity).

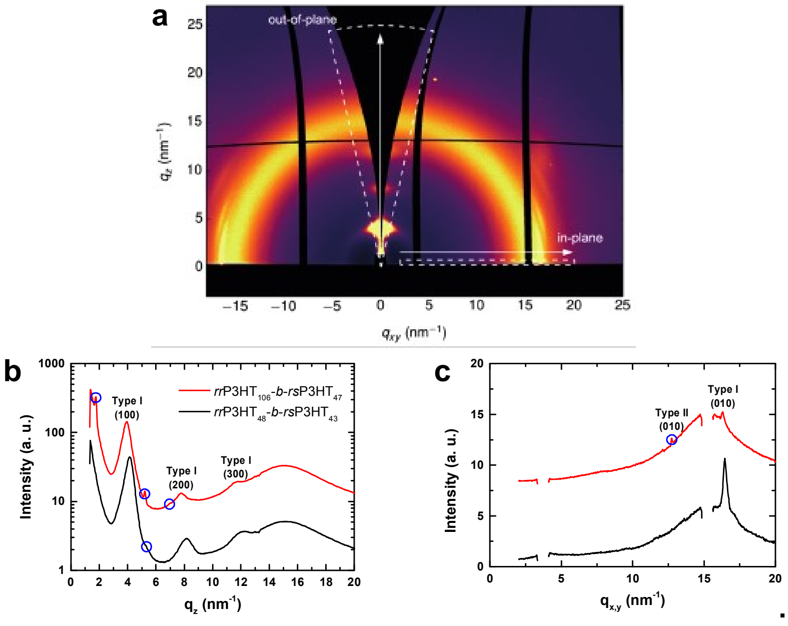

Supplementary Figure 7. GIWAXS of *rr*P3HT-*b*-*rs*P3HT fibers. Thin-film GIWAXS of *rr*P3HT$_{106}$-*b*-*rs*P3HT$_{47}$ and *rr*P3HT$_{48}$-*b*-*rs*P3HT$_{43}$ nanofibers in the form of (a) full 2D data image, (b) out-of-plane profile and (c) in-plane profile. Features belonging to the type II polymorph have been marked with blue circles.

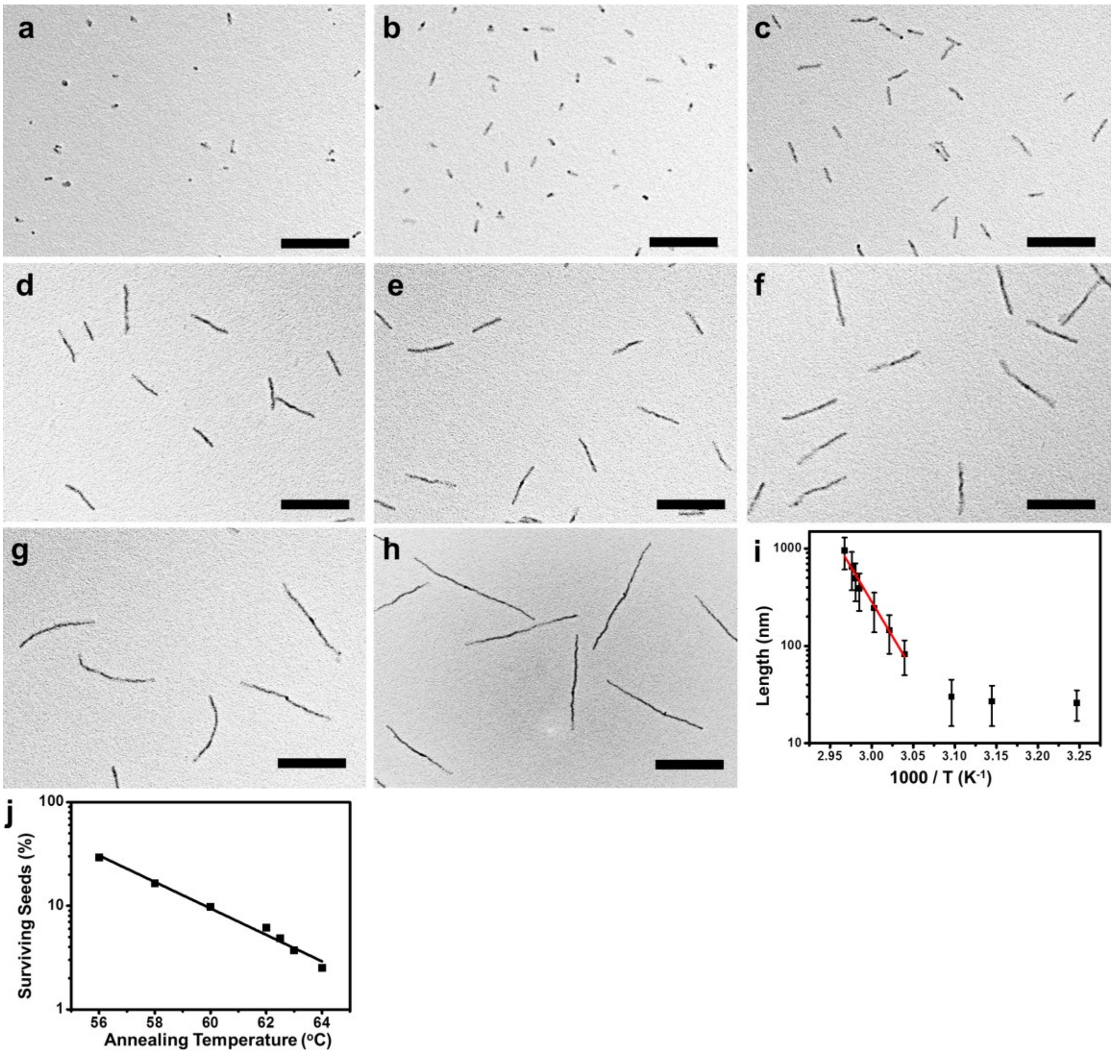

Supplementary Figure 8. TEM images and the corresponding contour length distribution of the *rr*P3HT$_{106}$-*b*-*rs*P3HT$_{47}$ fibers obtained in anisole via (a) sonication; and after thermal annealing at (b) 56 ^{o}C; (c) 58 ^{o}C; (d) 60 ^{o}C; (e) 62 ^{o}C; (f) 62.5 ^{o}C; (g) 63 ^{o}C; (h) 64 ^{o}C at 0.1 mg mL^{-1} for 1 h and cooling to 23 ^{o}C. Scale bars are 500 nm. (i) Semi-logarithmic plot of nanofiber length L_n versus 1000 / T [K^{-1}], where T is the dissolution temperature (from 35 ^{o}C to 64 ^{o}C). (error bars are the standard deviations σ for the length distribution). The straight line represents the best fit (from 56 – 64 ^{o}C) for the points of T to $log(L_n)$ = A-E/RT, where A is a constant. (j) Semi-logarithmic plot of fraction of surviving seeds in solution from *rr*P3HT$_{106}$-*b*-*rs*P3HT$_{47}$ vs annealing temperatures. The solid lines represent the best linear fits for the data points from 56.0 ^{o}C to 64.0 ^{o}C.

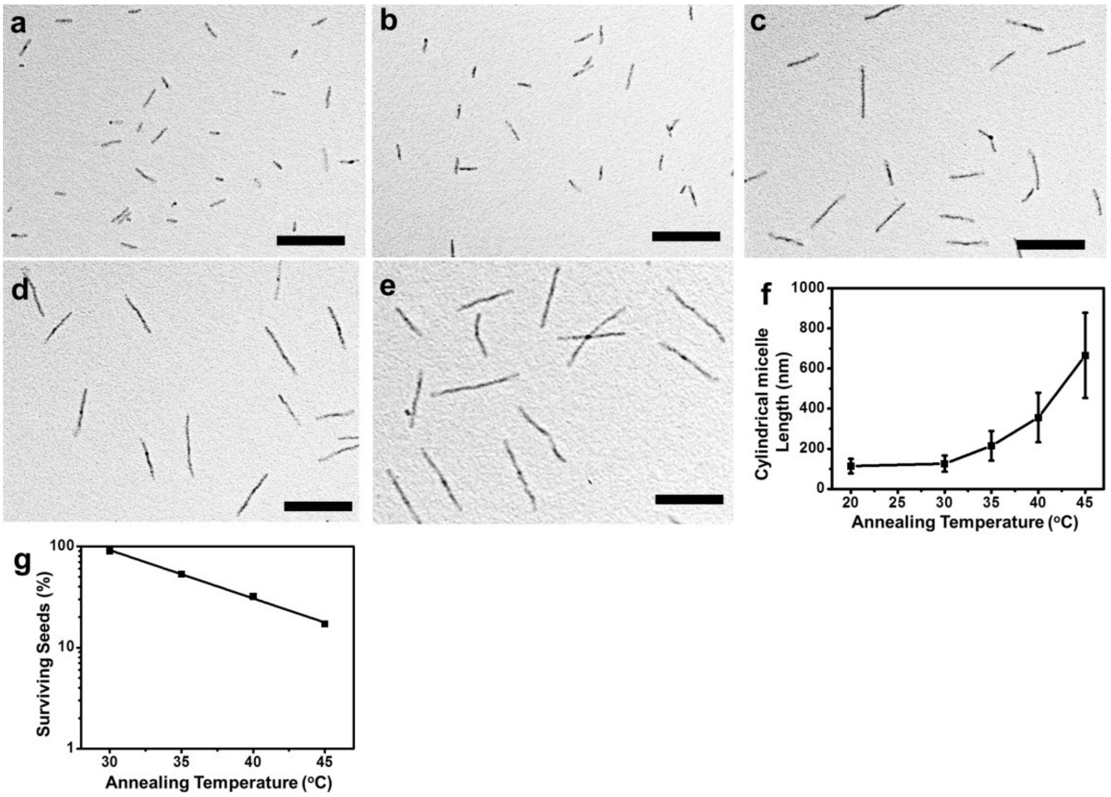

Supplementary Figure 9. TEM images of the *rr*P3HT$_{48}$-*b*-*rs*P3HT$_{43}$ fibers obtained in anisole via (a) sonication; and after thermal annealing at (b) 30 ^{o}C; (c) 35 ^{o}C; (d) 40 ^{o}C; and (e) 45 ^{o}C at 0.1 mg mL^{-1} for 1 h and then cooling to 23 ^{o}C. (f) Plot of the L_n of the *rr*P3HT$_{48}$-*b*-*rs*P3HT$_{43}$ fibers versus self-seeding temperatures. (g) Semilogarithmic plot of fraction of surviving seeds in solution (from *rr*P3HT$_{106}$-*b*-*rs*P3HT$_{47}$) vs annealing temperatures. The solid lines represent the best linear fits for the data points from 30 ^{o}C to 45 ^{o}C.

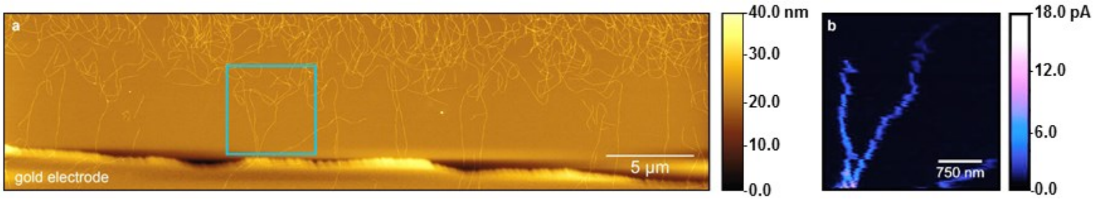

Supplementary Figure 10. (a) Large-area height image of the TUNA sample discussed in the main text (Fig. 2). The square indicates the approximate area imaged with the TUNA module in Fig. 2c. (b) Contact current TUNA image with the full current scale set to 17.5 pA.

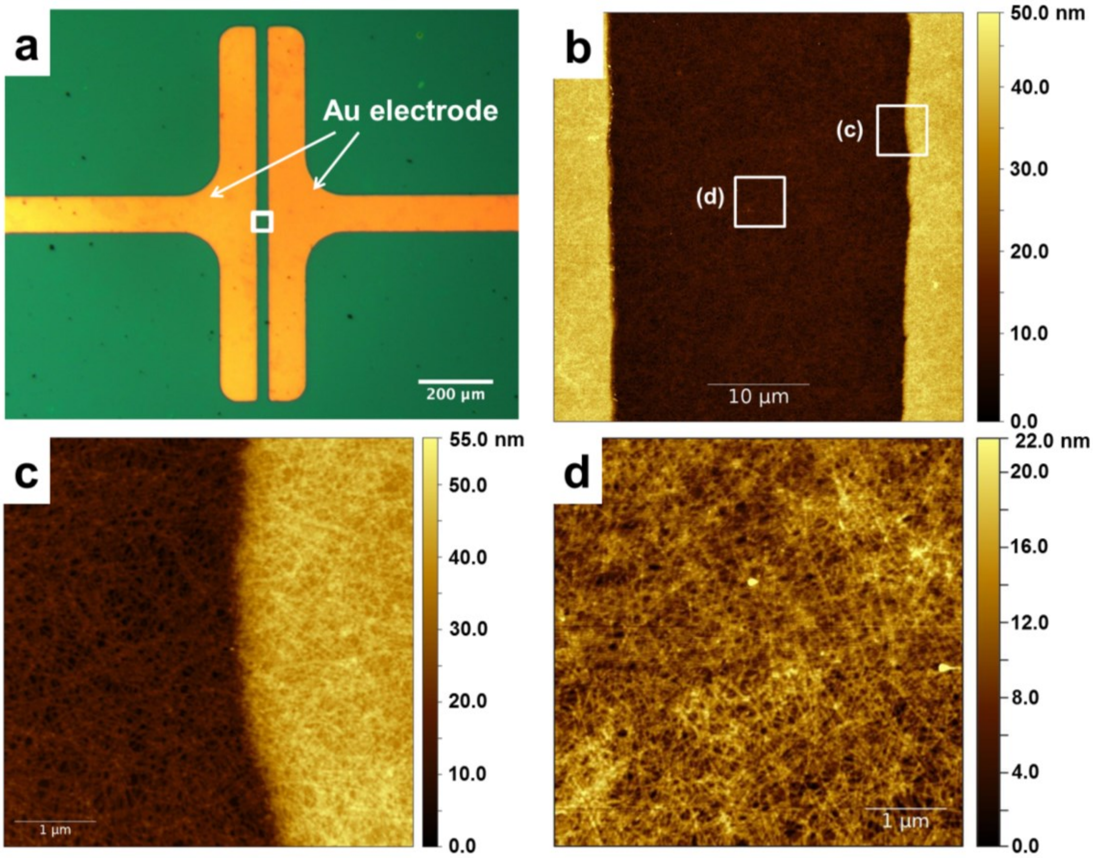

Supplementary Figure 11. (a) Optical micrograph the OFET devices used in this study. (b) Low resolution AFM images of the unfragmented *rr*P3HT$_{106}$-*b*-*rs*P3HT$_{47}$ fibers (length > 10μm) on the OFET device (AFM image obtained from the region within square as indicated in image (a)), (c, d) are the AFM images of unfragmented *rr*P3HT$_{106}$-*b*-*rs*P3HT$_{47}$ fibers at the (c) and (d) points in image (b).

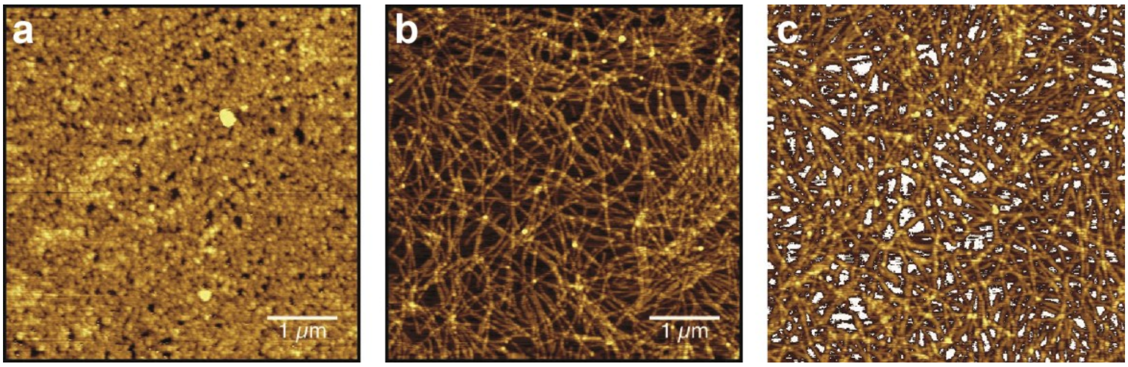

Supplementary Figure 12. AFM topographic images of the *rr*P3HT$_{106}$-*b*-*rs*P3HT$_{47}$ (a) seeds and (b) fibers prepared at 64.0 ^{o}C on OFET devices. The processed image in (c) shows the pixels considered to be empty space in (b), resulting in an estimated fill factor of 90%.

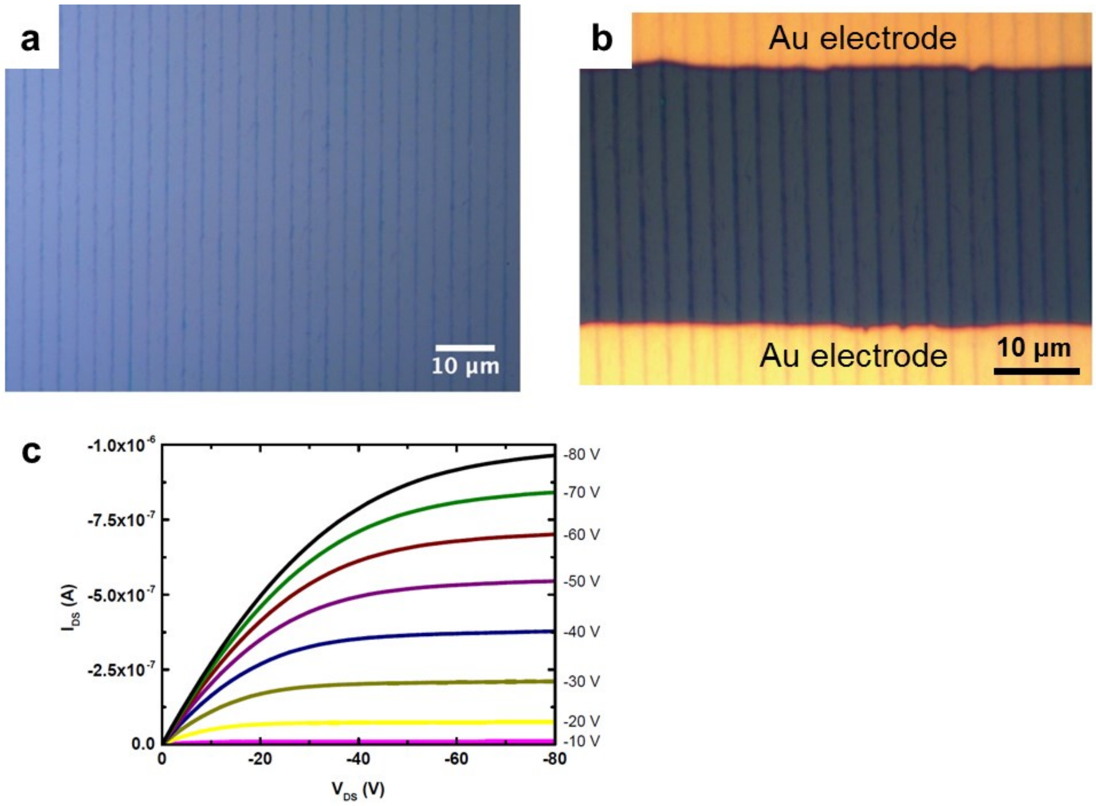

Supplementary Figure 13. Optical micrographs of (a) *rr*P3HT$_{106}$-*b*-*rs*P3HT$_{47}$ fibers deposited in the grooves of a lithographically photopatterned Si/SiO_2 substrate by dip-coating from 0.04 mg mL^{-1} dispersions in anisole. (b) Optical micrograph of a finished, top-contact *rr*P3HT$_{106}$-*b*-*rs*P3HT$_{47}$ transistor with a 30 µm channel length. (c) example output characteristics measured from a device with aligned *rr*P3HT$_{106}$-*b*-*rs*P3HT$_{47}$ fibers.

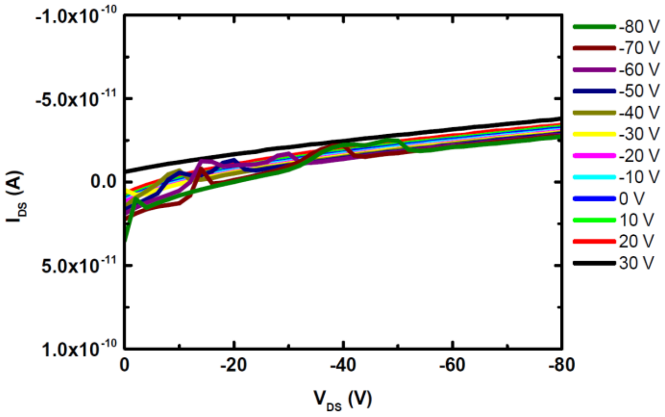

Supplementary Figure 14. Example output curves measured from a device from *rs*P3HT$_{90}$ homopolymer.

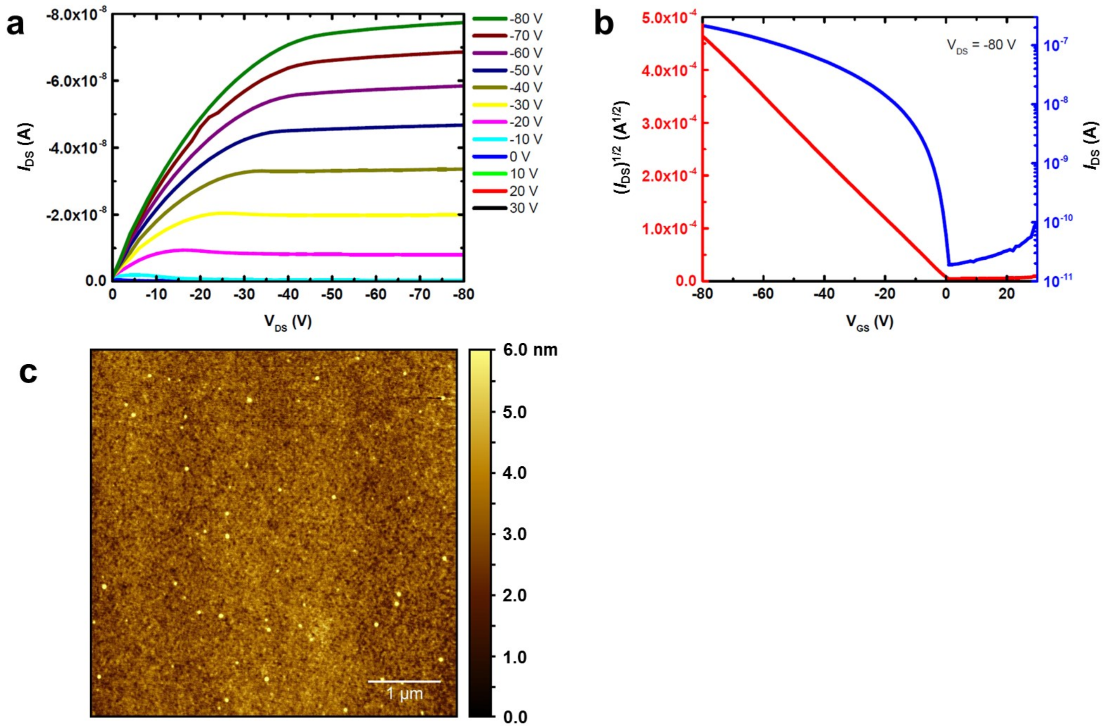

Supplementary Figure 15. Example (a) output and (b) transfer curves measured from a device from chloroform solution of *rr*P3HT$_{106}$-*b*-*rs*P3HT$_{47}$. Shown in (c) is the AFM image of the film obtained from the chloroform solution of *rr*P3HT$_{106}$-*b*-*rs*P3HT$_{47}$.

Supplementary Table 1| Molecular characteristics of both *rr*P3HT-*b*-*rs*P3HT and *rr*P3HT-*b*-PS diblock copolymers and their corresponding *rr*P3HT homopolymers.

	m/n [a]	M_n (KDa) [b]	M_n (KDa) [c]	M_w (KDa) [c]	PDI [c]
rr$P3HT_{48}$	N.A.	8.0	14.2	17.5	1.23
rr$P3HT_{106}$	N.A.	17.6	35.1	42.8	1.22
rr$P3HT_{54}$	N.A.	9.1	15.8	17.8	1.12
PS_{44}	N.A.	4.9	4.3	4.9	1.15
rs$P3HT_{90}$	N.A.	14.5	31.2	37.8	1.21
rr$P3HT_{48}$-*b*-*rs*$P3HT_{43}$	1.12	N.A.	31.4	37.7	1.20
rr$P3HT_{106}$-*b*-*rs*$P3HT_{47}$	2.23	N.A.	50.3	59.4	1.18
rr$P3HT_{54}$-*b*-PS_{44}	1.23	N.A.	22.5	25.3	1.12

[a] repeat unit number ratios of *rr*P3HT : *rs*P3HT, obtained from ^{1}H NMR spectroscopy;
[b] from MALDI-TOF mass spectrometry;
[c] from GPC analysis relative to polystyrene standard. The degree of polymerization (DP) of the *rr*P3HT homopolymers were calculated based on the results from MALDI-TOF and the block ratio was determined from ^{1}H NMR. The DP of the *rs*P3HT block was determined by combining these two results.

Supplementary Table 2| Characteristics of the monodisperse fibers prepared via self-seeding living CDSA method.

Annealing T (°C)	L_n (nm)	L_w (nm)	L_w / L_n	σ (nm)	σ / L_n
rr$P3HT_{106}$-*b*-*rs*$P3HT_{47}$ fibers					
23	24	27	1.13	8.6	0.36
35	26	29	1.12	9	0.35
45	27	31	1.19	12	0.44
50	33	38	1.15	13	0.39
56	82	94	1.15	32	0.39
58	145	172	1.18	62	0.43
60	245	292	1.19	107	0.44
62	390	457	1.17	162	0.41
62.5	494	581	1.18	207	0.42
63	647	763	1.18	274	0.42
64	952	1049	1.10	304	0.32
rr$P3HT_{48}$-*b*-*rs*$P3HT_{43}$ fibers					
20	114	125	1.10	36	0.32
30	126	139	1.10	40	0.32
35	215	240	1.12	74	0.34
40	356	398	1.12	123	0.35
45	665	733	1.11	212	0.32

Supplementary References

1. Ashiotis, G. *et al.* The fast azimuthal integration Python library: pyFAI. *J. Appl. Crystallogr.* **48,** 510–519 (2015).
2. Dane, T. A python library for reduction of 2-dimensional grazing-incidence and fibre diffraction data , https://github.com/tgdane/pygix, 2016.
3. Sirringhaus, H. Device physics of solution-processed organic field-effect transistors. *Adv. Mater.* **17,** 2411–2425 (2005).
4. Sirringhaus, H. Organic field-effect transistors: the path beyond amorphous silicon. *Adv. Mater.* **26,** 1319–1335 (2014).
5. Bittle, E. G., Basham, J. I., Jackson, T. N., Jurchescu, O. D. & Gundlach, D. J. Mobility overestimation due to gated contacts in organic field-effect transistors. *Nat. Commun.* **7,** 10908 (2016).
6. Sheina, E. E., Liu, J., Iovu, M. C., Laird, D. W. & McCullough, R. D. Chain growth mechanism for regioregular nickel-initiated cross-coupling polymerizations. *Macromolecules* **37,** 3526–3528 (2004).
7. Gwyther, J. *et al.* Dimensional control of block copolymer nanofibers with a π-conjugated core: crystallization-driven solution self-assembly of amphiphilic poly(3-hexylthiophene)-b-poly(2-vinylpyridine). *Chem. – A Eur. J.* **19,** 9186–9197 (2013).
8. Gill, R. E., Malliaras, G. G., Wildeman, J. & Hadziioannou, G. Tuning of photo- and electroluminescence in alkylated polythiophenes with well-defined regioregularity. *Adv. Mater.* **6,** 132–135 (1994).
9. Lee, S. W. et al. Temperature-dependent evolution of poly(3-hexylthiophene) type-II phase in a blended thin film. *Macromol. Rapid Comm.* **37**, 203–208 (2016).
